# Awareness and current implementation of drug dosage adjustment by pharmacists in patients with chronic kidney disease in Japan: a web-based survey

**DOI:** 10.1186/s12913-014-0615-0

**Published:** 2014-12-03

**Authors:** Yuki Kondo, Yoichi Ishitsuka, Eri Shigemori, Mitsuru Irikura, Daisuke Kadowaki, Sumio Hirata, Takeshi Maemura, Tetsumi Irie

**Affiliations:** Department of Clinical Chemistry and Informatics, Graduate School of Pharmaceutical Sciences, Kumamoto University, 5-1 Oe-honmachi, Chuo-ku, Kumamoto, 862-0973 Japan; Minaminihon Pharmaceutical Center, 5-15-1 Taniyama-chuo, Kagoshima, 891-0141 Japan; Laboratory of Evidence-Based Pharmacotherapy, College of Pharmaceutical Sciences, Daiichi University, 22-1 Tamagawa-cho, Minami-ku, Fukuoka, 815-8511 Japan; Department of Clinical Pharmacology, Faculty of Pharmaceutical Sciences, Kumamoto University, 5-1 Oe-honmachi, Chuo-ku, Kumamoto, 862-0973 Japan; Center for Clinical Pharmaceutical Sciences, Faculty of Pharmaceutical Sciences, Kumamoto University, 5-1 Oe-honmachi, Chuo-ku, Kumamoto, 862-0973 Japan

**Keywords:** Adjustment of drug dosage, Community pharmacists, Chronic kidney disease, Web-based questionnaire, pharmacy prescriptions

## Abstract

**Background:**

The aims of this study were to evaluate the current awareness of and implementation by pharmacists in Japan of adjustment of drug dosage according to renal function (ADDR) in patients with chronic kidney disease (CKD) and to clarify the factors influencing implementation of ADDR by community pharmacists.

**Methods:**

We conducted a web-based questionnaire of Japanese community and hospital pharmacists. Responders were compared by characteristics, rate of implementation of ADDR, experience with adverse drug events, pharmacist awareness of implementation of ADDR, and obstacles to ADDR implementation experienced by pharmacists. Additionally, the factors influencing the implementation of ADDR by community pharmacists were investigated by logistic regression analysis.

**Results:**

Fewer community pharmacists had implemented ADDR than hospital pharmacists. The community pharmacists had less experience with adverse drug events caused by an inappropriate dosage than the hospital pharmacists, while the hospital pharmacists had encountered more severe adverse drug events than the community pharmacists. The community pharmacists had less awareness of ADDR implementation, and believed that problems in implementing ADDR were caused by a lack of information on the renal function of patients. In the logistic regression analysis, the factors influencing implementation of ADDR were “Routinely receiving prescriptions from nephrologists”, “Experience with adverse drug events caused by inappropriate dosage for CKD patients”, and “Awareness of the need for pharmacists to check the dosage of renally excreted drugs”; they did not include “Lack of information on patient renal function”.

**Conclusions:**

This study indicates that fewer Japanese community pharmacists than hospital pharmacists implement ADDR and that implementation of ADDR by community pharmacists is hindered by their limited awareness of the importance of patient renal function. We advocate that many countermeasures be introduced to prevent CKD patients from experiencing adverse drug events caused by inappropriate dosage. Such countermeasures would include a training program to educate pharmacists about the impact of impaired renal function on dosage of drugs that are excreted by the kidneys.

**Electronic supplementary material:**

The online version of this article (doi:10.1186/s12913-014-0615-0) contains supplementary material, which is available to authorized users.

## Background

Chronic kidney disease (CKD) is, an important and common health problem. Its incidence and prevalence are increasing worldwide. The estimated overall prevalence of CKD (glomerular filtration rate <60 mL/min/1.73 m^2^) in adults (aged ≥18) is increasing exponentially in the older population in Japan [1]. Pharmacokinetics are often altered in CKD patients [2]. In particular, the optimal dosages of renally excreted drugs are strongly affected by renal impairment. Dosages that do not take renal function into account are a major cause of increases in drug blood concentrations that lead to adverse drug events [3,4]. Appropriate dosages of renally excreted drugs can be calculated on the basis of renal function using creatinine clearance. Thus, dosage adjustment based on renal function contributes to a reduction in the incidence of adverse drug events in older patients and others with renal impairment [5-7].

Adjustment of the drug dosage according to renal function (ADDR) by pharmacists, as a result of checking renal function and recommending alterations in their prescriptions, can prevent inappropriate dosages and thus reduce the incidence of the resulting adverse drug events [8,9]. Hassan et al. [6] reported that hospital pharmacists can contribute to a reduction in the incidence of adverse drug events in patients with renal impairment. Conversely, the contribution of community pharmacists to ADDR seems limited in Japan [10] and in other countries [11]. Although hospital pharmacists can easily obtain information on patient renal function from medical records, community pharmacists may find it difficult. We might expect that the limited contribution to the implementation of ADDR by community pharmacists might be because of the unavailability of information on patient renal function in community pharmacies. However, the practical reasons remain unclear. We conducted a questionnaire-based survey to evaluate the current awareness of community pharmacists of ADDR for CKD patients in Japan and to compare the responses of community pharmacists with those of hospital pharmacists. Additionally, to clarify why community pharmacists are not implementing ADDR, we explored factors influencing its implementation.

## Methods

### Study design

A web-based survey was developed to investigate various factors relating to the pharmacotherapy of CKD patients. A web-based questionnaire has advantages that include access to individuals in distant locations, the ability to reach difficult-to-contact participants, and the convenience of having automated data collection, which reduces researcher time and effort [12]. The survey was conducted via the Internet using “Google Forms”, a questionnaire-style information-collecting system for efficiently administering questionnaires [13]. The items in the survey are listed in Table 1. Pharmacists were invited to participate via the relevant pharmacist association’s mailing lists in each geographical area and via pharmacists’ groups on social networking sites. Because the URL of the survey website consisted of a random character string, general surfers of the Internet could not access the site. The timeframe for responding was 1 month (from May 1st to 31^st^, 2013). We did not send any reminders. The survey data were compared with Japanese national statistics on pharmacists from the Survey of Physicians, Dentists and Pharmacists 2012 by the Ministry of Health, Labour and Welfare [14]. We had estimated the percentage of pharmacists with <5 years working experience by the age distribution from the national statistics. The Ethics Committee of Kumamoto University approved the study (no. 788).

**Table 1:** **Questionnaire on pharmacotherapy for CKD patients**

**Information requested**	**Information obtained**
Type of work	Community pharmacist/Hospital pharmacist
Geographical distribution	Prefecture
Work experience (years)	<5, 5–9, 10–20, >20
Medical departments from which prescriptions are routinely received	Multiple choice format/17 departments (general internal medicine, respiratory medicine, gastroenterology, nephrology, endocrinology, hematology, psychiatry, pediatrics, orthopedics, dermatology, ophthalmology, urology, surgery, otorhinolaryngology, dentistry, obstetrics and gynecology)
Awareness of pharmacotherapy of CKD patients	Four items, using a 5-point Likert-type scale
Experience with adverse drug events caused by inappropriate dosage for CKD patients	Yes/No; when “yes” selected, respondent asked to name the drug that caused the adverse drug event
Implementation of ADDR	Implemented/not implemented
Obstacles to implementation of ADDR for CKD patients	Multiple choice format/five possible obstacles listed

### Statistical analysis

All statistical analyses were performed with EZR (Saitama Medical Center, Jichi Medical University, Japan), which is a graphical user interface for R [15] (The R Foundation for Statistical Computing, Vienna, Austria). More precisely, EZR is a modified version of R Commander designed to add statistical functions frequently used in biostatistics [16]. Univariate analyses to compare community and hospital pharmacists were performed using the chi-square or Mann–Whitney U tests. Logistic regression analysis was used to identify the factors influencing implementation of ADDR by community pharmacists. Logistic regression was performed using stepwise model selection according to the Akaike information criterion [17]. Significance values were set at *p* <0.05 for interpreting the final multivariate logistic regression model.

## Results

### Relevant characteristics of responders

Relevant characteristics of responders are shown in Table 2. Two hundred eighty-four pharmacists responded to the survey, including 190 community pharmacists (66.9%) and 94 hospital pharmacists (33.1%). Forty-five (15.8%), 73 (25.7%), 101 (35.6%), and 65 (22.9%) pharmacists had worked for <5, 5–9, 10–20, and >20 years, respectively. The community pharmacists routinely dispensed prescriptions for a median of three hospital departments. Eighty pharmacists (39.2%) routinely dispensed prescriptions from nephrologists. The community pharmacists routinely dispensed prescriptions from significantly fewer departments and dispensed significantly fewer prescriptions from nephrologists than the hospital pharmacists. The responding pharmacists were distributed throughout Japan (40/47 prefectures). The ratios of community to hospital pharmacists in the national statistics and in our data set were 2.5:1 and 3:1, respectively. The estimated percentage of pharmacists with <5 years of working experience in the national statistics was approximately 15.4%; the percentage with <5 years working experience in our dataset was similar (15.8%).

**Table 2:** **Characteristics of responders**

**Characteristic**	**Total**	**Community pharmacists**	**Hospital pharmacists**	***p*** **value**
Work experience, n (%)				0.63^a^
<5 years	45 (15.8)	27 (14.2)	18 (19.1)	
5–9 years	73 (25.7)	50 (26.3)	23 (24.5)	
10–20 years	101 (35.6)	71 (37.4)	30 (31.9)	
>20 years	65 (22.9)	42 (22.1)	23 (24.4)	
Number of departments for which prescriptions routinely dispensed, median (IQR)	3 (5)	3 (5)	4 (9)	<0.001^b^
Routinely dispense prescriptions from nephrologists, n (%)	80 (39.2)	33 (17.6)	47 (50.0)	<0.001^b^

^a^Chi-square test.

^b^Mann–Whitney test.

### Implementation of ADDR for CKD patients in Japan

We investigated the implementation of ADDR for CKD patients in Japan. Most hospital pharmacists (n = 86, 91.5%) implemented ADDR. In contrast, only about half of the community pharmacists (n = 103, 54.2%) had implemented ADDR (Table 3).

**Table 3:** **Rate of implementation of ADDR and experience with adverse drug events caused by inappropriate dosage for CKD patients**

**Questionnaire items**	**Community pharmacists n (%)**	**Hospital pharmacists n (%)**	***p*** **value**
Implementation of ADDR			<0.001^a^
Implemented	103 (54.2)	86 (91.5)	
Not implemented	87 (45.8)	8 (8.5)	
Experience with adverse drug events caused by inappropriate dosage for patient with CKD			<0.001^a^
Yes	22 (11.6)	47 (50)	
No	168 (88.4)	47 (50)	

^a^Chi-square test.

### Experience with adverse drug events caused by inappropriate dosage for CKD patients

**Figure 1:**
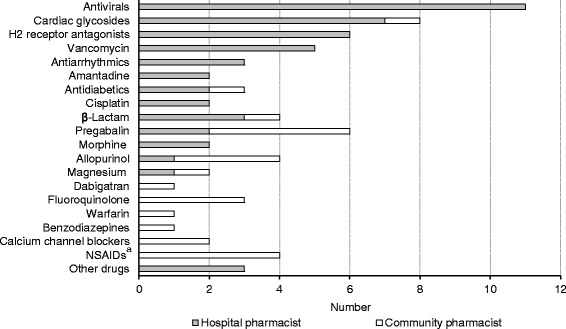
**Renally-excreted drugs cited by pharmacists as having caused ADRs.** ^a^NSAIDs:Non-steroidal anti-Inflammatory drugs.

We investigated the pharmacists’ experience with adverse drug events caused by an inappropriate dosage for CKD patients. As shown in Table 3, significantly fewer community pharmacists (n = 22, 11.6%) than hospital pharmacists (n = 47, 50.0%) had experienced such adverse drug events. We also investigated which drugs had caused these adverse drug events. The drugs most frequently cited by hospital and community pharmacists were antivirals and pregabalin, respectively. We also identified other differences between community and hospital pharmacists in terms of their experience with such adverse drug events (Figure 1).

### Awareness of pharmacotherapy for CKD patients

**Figure 2:**
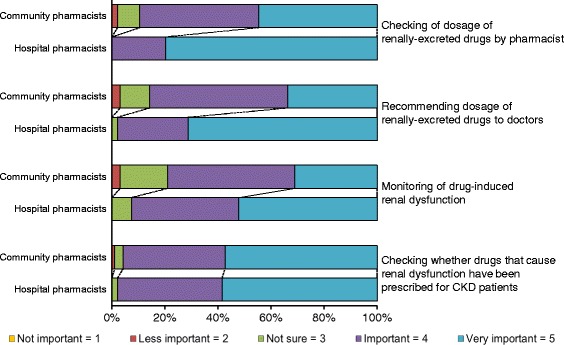
**Pharmacists’ awareness of pharmacotherapy for CKD in Japanese patients.** The awareness score is the median score on the Likert scale for each item.

We examined the participating pharmacists’ awareness of pharmacotherapy for CKD patients. Significantly fewer community pharmacists than hospital pharmacists were aware of the need for “Checking of dosage of renally excreted drugs”, “Recommending dosage of renally excreted drugs to doctors”, and “Monitoring of drug-induced renal dysfunction” (Median Likert scale (IQR) = 4 (1) to 5 (0), 4 (1) to 5 (1), and 4 (1) to 5 (1)). The score distributions are shown in Figure 2.

### Obstacles to ADDR implementation reported by pharmacists

As shown in Table 4, more community pharmacists (n = 168, 88.4%) than hospital pharmacists (n = 13, 13.8%) responded that “Difficulty in obtaining information on patient renal function” was an obstacle to implementation of ADDR.

**Table 4:** **Obstacles to ADDR implementation experienced by pharmacists**

**Characteristics**	**Community pharmacists n (%)**	**Hospital pharmacists n (%)**	***p*** **value**
Failure to check prescription because of pressure of other work	57 (30)	53 (56.4)	<0.001^a^
Difficulty in obtaining information on patient renal function (e.g., serum creatinine)	168 (88.4)	13 (13.8)	<0.001^a^
Fear of doctor’s rejection	32 (16.8)	10 (10.6)	0.227^a^
Lack of pharmacists’ skill	109 (57.4)	45 (47.9)	0.166^a^
Lack of relevant pharmaceutical information	61 (32.1)	42 (44.7)	0.052^a^

^a^Chi-square test.

### Factors influencing implementation of ADDR for CKD patients by community pharmacists

The final model of the logistic regression identified that the factors influencing implementation of ADDR by community pharmacists were “Routinely receiving prescriptions from nephrologists” (OR: 3.12, CI: 1.16–8.44), “Experience with adverse drug events caused by inappropriate dosage for CKD patients” (OR: 3.92, CI: 1.00–15.3), and “Awareness of need for pharmacists to check dosage of renally excreted drugs” (OR: 4.29, CI: 2.45–7.49) (Table 5).

**Table 5:** **Factors influencing implementation of ADDR by community pharmacists**

**Factor**	**Odds ratio**	**95% Confidence interval**	***p*** **value**
Routinely receive prescriptions from nephrologists			
No	(ref)	1.16–8.44	0.0247
Yes	3.12		
Experience with adverse drug events caused by inappropriate dosage			
No	(ref)	1.00–15.3	0.0498
Yes	3.92		
Work experience			
<5 years	(ref)	0.96–6.02	0.616
≥5 years	2.40		
Awareness of need for pharmacists to check dosage of renally excreted drugs	4.44	2.52–7.81	<0.001

Predictors: duration of work experience, Routinely dispense prescriptions from nephrologists, obstacle to implementation of ADDR, awareness of pharmacotherapy for CKD patients.

## Discussion

We demonstrated that community pharmacists had less awareness of and less frequently implemented ADDR for CKD patients than did hospital pharmacists. The less frequent implementation of ADDR by community pharmacists was attributable to differences in “Awareness of need for pharmacists to check dosage of renally excreted drugs”, “Routinely dispensing prescriptions from nephrologists”, and “Experience with adverse drug events caused by inappropriate dosage for CKD patients”.

Prescription errors, including inappropriate dosage for CKD patients, are associated with adverse drug events [18-21], and seem to be common. Via-Sosa et al. [11] and Joosten et al. [22] reported that community pharmacists sometimes dispense inappropriate dosages for CKD patients. In our study, half the participating community pharmacists had never implemented ADDR, and fewer community pharmacists than hospital pharmacists had implemented ADDR (Table 3). These findings suggest that community pharmacists sometimes dispense inappropriate dosages of drugs to CKD patients. Health care professionals’ awareness is influenced by their experience with clinical problems such as adverse drug events. In turn, the awareness of health care professionals, including pharmacists, greatly influences their behavior [23-25]. In our study, more hospital than community pharmacists had experienced adverse drug events (Table 3). Additionally, the hospital pharmacists had experienced more severe adverse drug events such as hospitalization and/or death than the community pharmacists (e.g., neurotoxicity of antivirals [26], digoxin intoxication [27]) (Figure 1). Fewer community pharmacists than hospital pharmacists were aware of the need to check and recommend appropriate dosages of renally excreted drugs and to monitor drug-induced renal dysfunction (Figure 2). Furthermore, in this study, pharmacists’ experience with adverse drug events was related to awareness of the need for checking and recommending appropriate dosages (data not shown). These findings suggest that hospital pharmacists have more experience with adverse drug events and that this results in them having more awareness of the need for ADDR. Thus, hospital pharmacists are more careful and are able to implement ADDR. In contrast, although many community pharmacists responded that checking the dosage was “important”, half of community pharmacists had not implemented ADDR. We believe there is a considerable gap between “important” and “very important”. Hence, we performed a subgroup analysis of the community pharmacists to compare awareness of the need for pharmacists to check dosage of renally excreted drugs and implementation of ADDR. Significantly more community pharmacists that had implemented ADDR responded that checking the dosage was “very important” rather than “important” (Additional file 1: Table S1). These results suggest a need for greater awareness among community pharmacists for implementing ADDR.

Most community pharmacists indicated that the most frequent obstacle to implementing ADDR was “Difficulty in obtaining information on patient renal function” whereas for most hospital pharmacists it was “Failure to check prescriptions because of pressure of other work” (Table 4). In Japan, community pharmacists generally do not have access to patient medical records, unlike hospital pharmacists. Thus, community pharmacists believe that their failure to implement ADDR is caused by a lack of information on patient renal function. However, about half the community pharmacists had implemented ADDR (Table 3). We therefore conclude that lack of information on renal function is not a critical factor in the failure of community pharmacists to implement ADDR.

To clarify the critical factors that influence implementation of ADDR by community pharmacists, we used logistic regression analysis. The final model of the logistic regression indicated that the influential factors were “Routinely dispensing prescriptions from nephrologists”, “Experience with adverse drug events caused by inappropriate dosage”, “Awareness of the need for pharmacists to check dosage of renally excreted drugs” (Table 5). However, “Difficulty in obtaining information about renal function” was not identified as a contributory factor. Additionally, in this study, dispensing prescriptions from nephrologists (i.e., participation in pharmacotherapy of CKD) tended to be related to awareness of the need for checking and recommending appropriate dosages (data not shown). These results are consistent with our argument that pharmacists’ experience with adverse drug events with CKD patients make them more likely to check prescriptions associated with ADDR policy. In contrast, in the Netherlands, the serum creatinine concentration is printed on prescriptions for designated drugs including renally excreted drugs (e.g., acyclovir) [28]. Recently, attempts to implement this policy have been made more frequently in Japan. However, although the serum creatinine concentration is generally an accurate indicator of renal function, this measure is less reliable in older people and those with malnutrition because of the loss of muscle mass [29-31]. Renal impairment is frequently overlooked in patients with such concealed renal impairment (normal serum creatinine concentrations and reduced estimated glomerular filtration rate), resulting in the prescription of inappropriate dosages [32]. Wong et al. [33] stressed that awareness that renal impairment can be accompanied by normal serum creatinine concentrations is important in preventing adverse drug events in older patients. These findings suggest that implementation of ADDR by community pharmacists would be best supported by not only providing information about serum creatinine concentrations, but also by the strategy of bringing renal impairment to the attention of pharmacists when they check prescriptions, by means, for example, of a computerized system that alerts the pharmacist to renal impairment. Joosten et al. [22] have demonstrated that a computer-based renal impairment alert system is effective. However, it would be difficult to introduce a computerized alert system for all community pharmacists. Conversely, training programs for physicians [7], medical staff [34], and community pharmacists [11] seem to reduce the percentage of inappropriate dosage prescriptions. Thus, training programs to educate community pharmacists on the risk of adverse drug events caused by inappropriate dosages for CKD patients and encouraging them to pay attention to renal impairment may be effective in increasing the rate of implementation of ADDR.

This study has some limitations. Data validation in this survey was not sufficient. Although we compared our data with national statistics, this survey data lacks some demographic characteristics of respondents, such as gender, age, and qualifications of the pharmacist. Furthermore, the questionnaire invitation was sent via a closed social network group and relevant pharmacist associations’ mailing lists. Because the exact number of invitees was unknown, we were not able to calculate a response rate and sample size. Additionally, this study has a sampling bias, which is a disadvantage of the survey method. A web-based questionnaire also has a self-selection/volunteer bias [35]. This survey might have been biased toward pharmacists who are concerned with or have an interest in ADDR. Thus, this study might have overestimated the percentage of pharmacists who have implemented ADDR. Conversely, a web-based survey is easier for participants to respond to because pharmacists who have not implemented ADDR might feel stigmatized if the survey were public [12]. Therefore, we chose the web-based approach as a means of obtaining more responses from pharmacists in Japan. Although further study will be needed, the results suggest the importance of awareness among pharmacists in implementing ADDR.

## Conclusions

In summary, we have investigated the awareness and implementation of ADDR for CKD patients by community pharmacists. We found that community pharmacists commonly adjust inappropriate dosages of drugs for CKD patients. However, there is insufficient implementation of ADDR by community pharmacists and insufficient awareness of the need for community pharmacists to check prescriptions for patients with renal impairment and to implement ADDR. We believe that immediate measures such as training programs for community pharmacists are necessary to improve the current status of ADDR implementation in Japan by drawing their attention to the need to check prescriptions for patients with renal impairment.

## Additional file

Additional file 1: Table S1. Relationship between awareness of need for pharmacists to check the dosage of renally excreted drugs and implementation of ADDR by community pharmacists.

## Abbreviations

CKD: Chronic kidney disease
ADDR: Adjustment of drug dosage according to renal function
