# Correction to: Awareness and current implementation of drug dosage adjustment by pharmacists in patients with chronic kidney disease in Japan: a web-based survey

**DOI:** 10.1186/s12913-021-06631-w

**Published:** 2021-06-29

**Authors:** Yuki Kondo, Yoichi Ishitsuka, Eri Shigemori, Mitsuru Irikura, Daisuke Kadowaki, Sumio Hirata, Takeshi Maemura, Tetsumi Irie

**Affiliations:** 1 Department of Clinical Chemistry and Informatics, Graduate School of Pharmaceutical Sciences, Kumamoto University, 5-1 Oe-honmachi, Chuo-ku, Kumamoto, 862-0973 Japan; 2 Minaminihon Pharmaceutical Center, 5-15-1 Taniyama-chuo, Kagoshima, 891-0141 Japan; 3 Laboratory of Evidence-Based Pharmacotherapy, College of Pharmaceutical Sciences, Daiichi University, 22-1 Tamagawa-cho, Minami-ku, Fukuoka, 815-8511 Japan; 4 Department of Clinical Pharmacology, Faculty of Pharmaceutical Sciences, Kumamoto University, 5-1 Oe-honmachi, Chuo-ku, Kumamoto, 862-0973 Japan; 5 Center for Clinical Pharmaceutical Sciences, Faculty of Pharmaceutical Sciences, Kumamoto University, 5-1 Oe-honmachi, Chuo-ku, Kumamoto, 862-0973 Japan

**Correction to: BMC Health Serv Res 14, 615 (2014)**

**https://doi.org/10.1186/s12913-014-0615-0**

Following publication of the original article [1], the authors identified an error in the column ***p*** **value** in Table 5. They erroneously listed the *p*-value for working experience as "0.616", but the correct value is "0.0616". The correct table is given below.

**Table 5:** Factors influencing implementation of ADDR by community pharmacists

Factor	Odds ratio	95% Confidence interval	***p*** value
Routinely receive prescriptions from nephrologists			
No	(ref)	1.16–8.44	0.0247
Yes	3.12		
Experience with adverse drug events caused by inappropriate dosage			
No	(ref)	1.00–15.3	0.0498
Yes	3.92		
Work experience			
<5 years	(ref)	0.96–6.02	0.0616
≥5 years	2.40		
Awareness of need for pharmacists to check dosage of renally excreted drugs	4.44	2.52–7.81	<0.001

Predictors: duration of work experience, Routinely dispense prescriptions from nephrologists, obstacle to implementation of ADDR, awareness of pharmacotherapy for CKD patients

The original article [1] has been updated.
