# Supplementary material for: Awareness and current implementation of drug dosage adjustment by pharmacists in patients with chronic kidney disease in Japan: a web-based survey
Source: BMC Health Serv Res. 2014 Dec 3;14:615. doi: 10.1186/s12913-014-0615-0 (PMC4258280; doi:10.1186/s12913-014-0615-0)
Supplement: Additional file 1: Table S1. — Relationship between awareness of need for pharmacists to check the dosage of renally excreted drugs and implementation of ADDR by community pharmacists. [file 12913_2014_615_MOESM1_ESM.docx]

| **Supplemental Table 1: Relationship between awareness of need for pharmacists to check the dosage of renally excreted drugs and implementation of ADDR by community pharmacists.** | | | | |
| --- | --- | --- | --- | --- |
| **Questionnaire items** | **Awareness of need for pharmacists to check dosage of renally-excreted drugs** | |  |  |
|  | **Important**  **(score=4)** | **Very important (score=5)** | ***p* value** |  |
| Implementation of ADDR |  |  |  |  |
| Implemented (%) | 32 (37.6) | 67 (78.8) | <0.001 ^a^ |  |

^a^ Chi-square test.
